# Comprehensive Analysis of Magnetic Flux Density and RF-EMF Exposure in Electric Buses: A Case Study from Samsun, Turkey

**DOI:** 10.3390/s24175634

**Published:** 2024-08-30

**Authors:** Zafer Emre Albayrak, Cetin Kurnaz, Teoman Karadag, Adnan Ahmad Cheema

**Affiliations:** 1 Mechanical, Electrical, and Lighting Branch, Department of Public Works, Samsun Metropolitan Municipality, Samsun 55200, Türkiye; 2 Department of Electrical and Electronic Engineering, Ondokuz Mayıs University, Samsun 55139, Türkiye; ckurnaz@omu.edu.tr; 3 Department of Electrical and Electronic Engineering, Inonu University, Malatya 44280, Türkiye; teoman.karadag@inonu.edu.tr; 4 School of Engineering, Ulster University, Belfast BT15 1AP, UK; a.cheema@ulster.ac.uk

**Keywords:** magnetic flux density, radiofrequency electromagnetic field, electric buses, public transportation, personal exposure, health risks, ICNIRP

## Abstract

This study investigates magnetic flux density (B) and radiofrequency electromagnetic field (RF-EMF) measurements on electric buses operating in Samsun, Turkey, focusing on two bus routes (called E1 and E4) during the morning and evening hours. Measurements were taken under diverse operational conditions, including acceleration, cruising, and braking, at locations of peak passenger density. Along the E1 route, the magnetic field intensity varied significantly based on the bus position, road slope, and passenger load, with notable increases during braking. In contrast, the E4 route showed a lower magnetic field intensity and RF-EMF values due to its straighter trajectory and reduced operational stops. The highest RF-EMF measurement recorded was 6.01 V/m, which is below the maximum levels established by the ICNIRP guidelines. In 11 out of the 12 different band-selective RF-EMF measurements, the highest contribution came from the downlink band of the base stations, while in only one measurement, the highest contribution originated from the uplink bands of the base stations. All data were subject to the Anderson–Darling test, confirming the generalized extreme value distribution as the best fit for both B and RF-EMF measurements. Additionally, the study assessed B levels inside and outside the bus during charging, revealing heightened readings near the pantograph. These findings significantly contribute to our understanding of electromagnetic field exposure in electric bus environments, highlighting potential health implications and informing the development of targeted mitigation strategies.

## 1. Introduction

Electromagnetic fields (EMFs) are integral to modern life, spanning frequencies from an extremely low frequency (ELF) to radiofrequency (RF) and beyond. Low-frequency EMF sources, such as power lines and household appliances, operate in the 50–60 Hz range, while high-frequency sources, like mobile phones and Wi-Fi, operate in the MHz to GHz range. The widespread use of these technologies has raised public concerns about potential health effects, leading to extensive research on EMFs’ biological impacts. Several studies have examined the health effects of radiation from various sources, particularly focusing on mobile phone base stations and personal exposure in different environments. For example, Röösli et al. conducted a comprehensive review of the health effects of radiation from mobile phone base stations, summarizing the current literature and emphasizing the ongoing debate about potential health risks [1]. Joseph et al. compared personal radiofrequency electromagnetic field (RF-EMF) exposure levels across different European countries and found that, while RF-EMF exposure levels were generally well below international limits, the highest exposure occurred inside transport vehicles such as trains, cars, and buses [2]. Joseph et al. characterized the general public’s EMF exposure from 12 different radiofrequency sources, indicating that indoor exposure in office environments can be higher than outdoor exposure, with the highest total field exposure occurring in mobile scenarios such as inside trains or buses [3]. In the study by Viel et al., residential radiofrequency exposure from antennas was assessed using personal exposure meters, revealing that Global System for Mobile Communication (GSM) and digital communication system waves have higher exposure levels closer to antennas, peaking at specific distances due to their complex propagation characteristics, which underscores the importance of considering distance when evaluating RF exposure near base stations [4]. Kühn et al. examined radiation emissions from mobile phones used with both wireless and wired hands-free kits to assess compliance with safety standards, underscoring the need for ongoing evaluations of exposure limit compliance [5]. The study by Foster and Trottier reviewed the effects of RF on human health, highlighting significant methodological challenges and gaps in existing research and emphasizing the need for more rigorous studies to understand better the long-term health implications of RF-EMF exposure [6]. The study by Aerts et al. finds that RF-EMF exposure from 5G small cells is well within the International Commission on Non-Ionizing Radiation Protection (ICNIRP) limits, with maximum exposure ratios of 0.15 for occupational exposure and 0.68 for the general public, while a significantly lower level of exposure is observed for non-users depending on the base station’s activity and beam-forming capabilities [7]. The study by Chiaraviglio et al. reveals that in 5G mobile networks, EMF exposure from smartphones during uplink services is more dominant than that from radio base stations [8]. Qin et al. showed that when user density significantly exceeds base station density, passive EMF exposure from other users’ devices is comparable to active EMF exposure from both base stations and the user’s device [9]. Adda et al. found that 6 min measurements taken during peak traffic on working days may overestimate daily exposure levels and proposed an extrapolation factor to estimate average daily exposure from short-term data [10]. Mulugeta et al. demonstrated that indoor RF-EMF downlink exposure from distant outdoor cellular antennas can be modeled with a Gaussian distribution based on 1176 measurements in three buildings, all showing exposure levels well below 1% of ICNIRP reference levels [11].

Governments are increasingly adopting electric vehicles (EVs) in public transportation to address the challenges of declining fossil fuel resources and rising environmental pollution. EVs offer lower energy costs and zero emissions, making them a promising alternative to fossil-fueled vehicles. As technology advances, electric buses are replacing diesel ones in urban transport, supported by charging point installations. However, while EVs reduce CO_2_ emissions, they also emit EMFs that can impact passengers and drivers. In public transport, individuals are exposed to EMFs from high-power electrical systems and various wireless communication technologies, including Wi-Fi routers inside vehicles, mobile communication handsets, and base transceiver stations outside vehicles. This raises concerns about chronic EMF exposure, but research on this issue in public transportation remains limited. Understanding and monitoring EMF levels in electric buses are essential for ensuring safety and regulatory compliance, as well as minimizing potential health impacts, ultimately contributing to safer and more sustainable urban environments.

Various studies in the literature examine the impact of EMFs generated in public transport vehicles on public health, as summarized in Table 1. Judokova and Janousek reviewed EMF sources in transport and their potential health effects [12]. Halgamuge et al. measured electric and magnetic field levels in and around trains, trams, and hybrid vehicles at various locations and times in Melbourne [13]. In the study by Abbasov et al., magnetic field measurements were taken along the tram bus route in Malatya, covering a frequency range of 1 Hz to 400 kHz [14]. Echarri et al. used simulations to calculate electric field distributions from 2G and 5G communication technologies in Bilbao public transport trams, analyzing passenger exposure levels [15]. The study by Hristov et al. conducted electric and magnetic field measurements in various electric and hybrid vehicles in Bulgaria, comparing the levels to Bulgarian standards [16]. Bae and Park measured electric and magnetic field levels from six different EV chargers and compared them to ICNIRP standards [17]. Lee et al. measured EMF levels in and around a train equipped with a 1 MW wireless charging system in Korea, comparing the results to ICNIRP standards through a simulation [18]. The study by Echarri et al. measured and analyzed electric fields in buses and subways in Pamplona, Spain, according to ICNIRP standards [19]. Gryz et al. measured electric and magnetic field values at and around charging locations for urban transport vehicles across various sites [20]. Havas et al. measured magnetic flux densities in the passenger compartments of buses, trams, subways, and trains in Toronto [21]. Tell et al. measured electric and magnetic field values around and inside a bus being wirelessly charged in Chattanooga [22]. The study by Chadwick and Lowes presented magnetic flux density (B) measurement results from electric trains and trams in the UK [23]. Finally, the study by Eraydin et al. measured EMF radiation from Wi-Fi equipment on the Samsun Light Rail System over five days, finding that the levels were below ICNIRP’s reference level [24].

Moreno-Torres et al. evaluated the electromagnetic environment within EVs to assess the potential hazards of passenger exposure to magnetic fields. Their study emphasized the importance of incorporating electromagnetic safety considerations into vehicle design using estimation tools based on finite element simulation [25]. Dong et al. evaluated passengers’ EMF exposure in various seated positions on a pure electric AC bus. Their study found that while the B and induced electric field intensity were generally below ICNIRP limitations, some instances of elevated electric field intensity on the passengers’ scalps highlighted the need for the further development of electromagnetic shielding in vehicle design [26]. Li Gang et al. analyzed the low-frequency magnetic field distribution in an EV cabin and assessed the safety of human exposure through a simulation analysis. Their study found that the test results were below the safety limits set by standards and provided theoretical guidance for low-frequency magnetic field protection in vehicle design by offering insights into the effects of frequency, driving current, vehicle body material, and wiring layout on induced EMFs [27].

### 1.1. Research Gaps of Previous Works for Magnetic Field and RF-EMF Assessments

Research on EMF exposure from electric buses can raise public awareness and help develop necessary precautions, enriching fields such as health, regulation, environment, and technology and serving as a valuable guide for future research efforts. However, previous studies have focused on magnetic or RF-EMF measurements separately without comprehensively assessing both EMFs in electric bus environments. Research has been constrained by small sample sizes or has been limited to specific bus routes, thus restricting the generalizability of findings. Some studies lacked adequate spatial coverage, failing to capture EMF level variations at different bus locations. Similarly, temporal coverage has been insufficient, and variations in EMF levels at different times of the day or operational conditions have been neglected. Previous research has also omitted comparisons of EMF exposure levels between electric buses and conventional diesel or petrol-powered buses, thereby overlooking potential differences in exposure between bus types. Additionally, inadequate consideration has been given to the impact of passenger density on EMF exposure levels, resulting in an oversight of differences in exposure between crowded and empty buses. Finally, the influence of the charging process on B, particularly near critical components like the pantograph, has not been thoroughly investigated.

In response to the identified research gaps, this study was designed to address the limitations of previous works comprehensively. We took extensive B and RF-EMF measurements across various bus environments in Samsun, Turkey to ensure a holistic assessment of EMF exposure. Our study expanded its spatial coverage by including multiple bus routes across different districts, capturing EMF variations at various bus locations. We also improved temporal coverage by taking measurements at different times of the day, providing a dynamic understanding of EMF levels.

Our research includes a comparative analysis of EMF exposure between electric buses and conventional diesel-powered buses, highlighting potential differences in exposure levels. We specifically evaluated how passenger density affects EMF exposure, addressing the critical factor of crowding inside buses. Furthermore, the study explored the impact of the charging process, particularly near critical components like the pantograph, on B measurements.

To strengthen the accuracy of our analysis, we performed Anderson–Darling goodness-of-fit tests for several statistical distributions. This testing aimed to identify the most appropriate distribution models for the measured B and RF-EMF levels. By applying this rigorous method, we ensure that our findings more accurately represent the underlying distribution characteristics of the data, leading to a clearer and more precise understanding of EMF exposure in electric bus environments.

By explicitly structuring the methodology and results sections to reflect these objectives, this study provides a detailed and holistic assessment of EMF exposure in electric bus environments. The findings offer valuable insights for future research and contribute significantly to public health, transportation safety, and EMF regulation, ensuring a safe and sustainable transition to electric public transportation.

### 1.2. Contributions of This Paper

The main contributions of this study can be summarized as follows:

- Detailed EMF Exposure Assessment: Provides a comprehensive analysis of EMF exposure levels experienced by drivers and passengers in electric buses, considering various conditions such as different bus environments, times of the day, and passenger densities;
- Case Study in Samsun, Turkey: Provides a detailed assessment of B and RF-EMF exposure in electric buses in Samsun, Turkey. This case study highlights specific regional health risks and exposure characteristics, offering valuable insights into local public health and transportation safety;
- Comparative Analysis: Includes a comparative analysis of EMF exposure between electric and conventional diesel-powered buses, revealing potential differences in exposure levels and providing a clearer understanding of how different bus types affect EMF exposure;
- Impact of Passenger Density and Charging Process: Evaluates how passenger density influences EMF exposure and explores the effects of the charging process, particularly near critical components like the pantograph, on B;
- Statistical Analysis: To enhance the accuracy of our findings, we conducted Anderson–Darling goodness-of-fit tests to identify the best-fitting distribution models for the measured B and RF-EMF levels. This approach ensures a clearer and more precise understanding of EMF exposure in electric bus environments;
- Guidance for Safety and Policy: Identifies research gaps and informs strategies for mitigating EMF exposure, enhancing public transportation safety, and guiding future research and policymaking on EMF safety regulations. The findings contribute to refining existing EMF regulations and offer guidance for developing new policies;
- Future Research Directions: Highlights the need for future studies to explore the long-term health effects of EMF exposure and the potential for further optimizing safety measures in electric buses.

## 2. Materials and Methods

### 2.1. Reference Levels for Magnetic and Electric Fields

In many countries, specific regulations and standards govern exposure to EMFs. The effects of electric and magnetic fields on human health have been a significant research focus for many years, as potential health implications from exposure are a concern in industry, medicine, and daily life. Numerous countries have established regulations and standards for exposure to low-frequency magnetic and high-frequency EMFs. The ICNIRP plays a crucial role in evaluating the effects of electric and magnetic fields on human health and establishing limit values. These ICNIRP limit values, grounded in scientific research, aim to minimize potential risks associated with daily electric and magnetic field exposure. They serve as general guidelines for managing and controlling exposure levels to these fields, applicable to occupational and general public settings.

**Figure 1:**
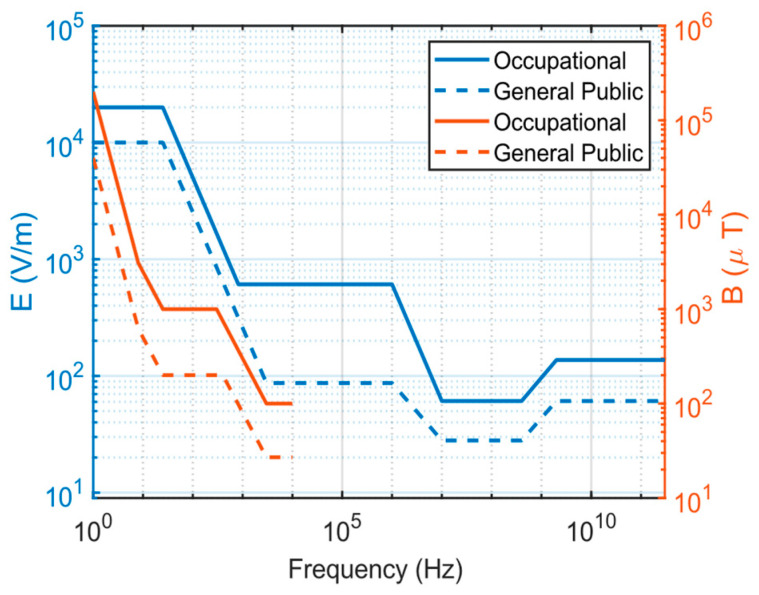
ICNIRP reference levels for electric and magnetic fields for occupational and general public radiation exposure with frequency dependency.

Figure 1 presents frequency-dependent ICNIRP reference levels for B [28] and electric fields [29,30] regarding occupational and general public radiation exposure. For occupational exposure to B, the reference levels are 200,000 μT for 1 Hz frequencies, 1000 μT for 50 Hz, 300 μT for 1 kHz, and 100 μT for 400 kHz. Correspondingly, for the general public, these values are set at 40,000 μT, 200 μT, 80 μT, and 27 μT, respectively [28]. For the frequency range between 80 MHz and 6 GHz, which is the focus of our study, the limit values range from 61 V/m to 137 V/m for occupational exposure and from 28 V/m to 61 V/m for general public exposure. Concerning base stations operating at specific frequencies, the limit values for occupational exposure to electric field strength are as follows: 84.385 V/m for 800 MHz, 90 V/m for 900 MHz, 127.2 V/m for 1800 MHz, 137 V/m for 2100 MHz, and 2600 MHz. For the general public, ICNIRP has determined limit levels of 38.8 V/m, 41.2 V/m, 58.3 V/m, 61 V/m, and 61 V/m, respectively, for base stations operating at 800 MHz, 900 MHz, 1800 MHz, 2100 MHz, and 2600 MHz [29,30].

### 2.2. Magnetic Field and RF-EMF Measurement Campaign

**Figure 2:**
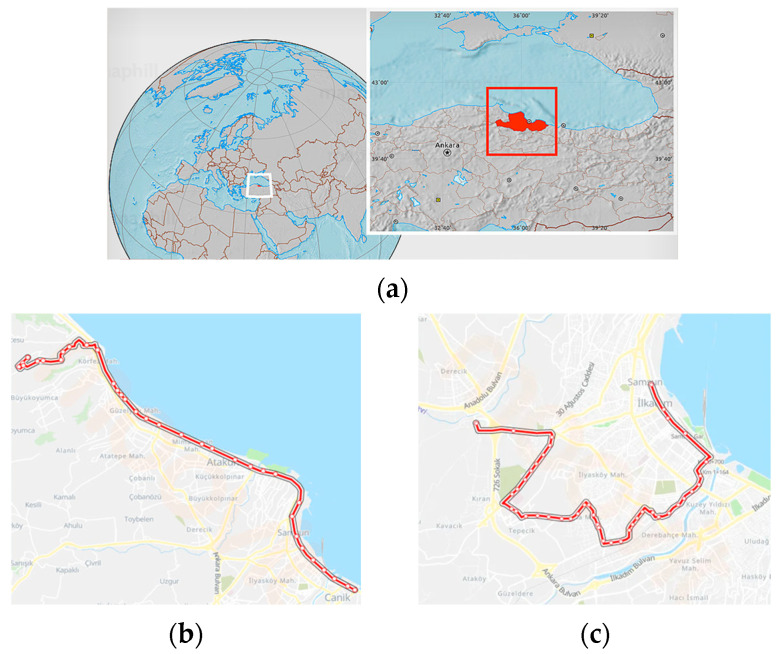
(**a**) Samsun city (the white box indicates Turkey, and the red box indicates the city of Samsun), (**b**) E1, (**c**) E4 electric bus routes.

Samsun Metropolitan Municipality (SMM) is one of the municipalities in Turkey that has transitioned to using EVs for urban public transportation, replacing diesel-fueled vehicles. By August 2022, SMM had acquired 20 electric buses, named Avenue, which boast zero-emission values. These buses were manufactured through collaboration between TEMSA and ASELSAN and were deployed on the E1 and E4 routes [31]. The routes for the E1 and E4 lines are depicted in Figure 2. The E1 bus route comprises 35 stops from Soguksu station to OMU Faculty of Education station, covering a total length of 24.7 km. The total journey time for this route is approximately 52 min. The E4 bus route includes 43 stops from Otogar station to Büyük Cami station, spanning 13.3 km. The total journey time for this route is approximately 34 min. The E1 bus route passes through the Canik, İlkadım, and Atakum districts of Samsun, while the E4 bus route is located only in the İlkadım district. The population density of the Canik district is 380/km^2^, that of İlkadım district is 2200/km^2^, and that of Atakum district is 690/km^2^ [32]. The maximum speed of the buses is 50 km/h, although it can drop to 30 km/h in special areas such as schools and for pedestrian crossings.

**Figure 3:**
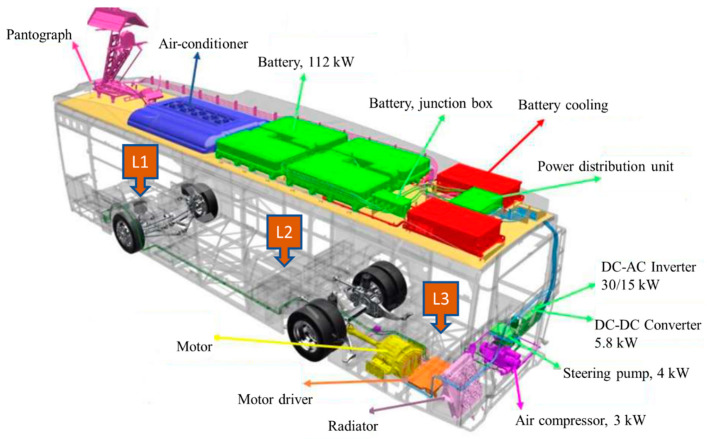
The general structure of the electric bus used in the measurements.

Detailed information about these electric buses, which are produced in cooperation with TEMSA and ASELSAN and used on the E1/E4 routes, is provided in Figure 3. The buses are equipped with four modular and parallel-connected batteries totaling 90 kW of power, positioned on the bus’s roof. These batteries are charged with 650 V DC, with cables from each battery individually connected to the power distribution box. From there, the cables lead to the motor drive, where the 650 V DC is converted into 600 V AC. This AC powers the 275 kW AC motor located at the bus’s rear, transferring electrical energy to the bus wheels. Additionally, the bus features a battery cooling system, a compressor operating with 600 V AC in the rear section, fans operating with 24 V DC in the upper section, and a pump operating with 24 V DC in the rear section. Utilizing pantographs on the bus’s roof, an 80% charge can be achieved in 15 min, with a full charge (100%) attained in 30 min. When fully charged, the buses can travel up to 80 km. The dimensions of the electric bus are as follows: length, 12 m; width, 2.55 m; and height, 3.654 m. The total passenger capacity of the vehicle is 79, comprising 35 seated passengers, 43 standing passengers, and one disabled passenger.

This study measured electric and magnetic field values at various locations in and around electric buses deployed on the E1 and E4 routes. To assess exposure to EMFs, three measurement points were identified inside the bus, considering the primary sources of EMF emissions. The goal was to determine the radiation levels to which drivers and passengers are exposed. Measurements were taken at three distinct positions within each bus: the front, center, and rear. Specifically, these positions were the driver’s side (denoted as L1), the vehicle’s center (L2), and the vehicle’s rear (L3), as illustrated in Figure 3. This approach aimed to ascertain the magnetic and electric field intensities at different positions within the bus. Measurements on the E1 and E4 routes were taken during peak passenger times in the morning (07:00–09:00) and evening (17:00–19:00). The E and B field measurements were taken simultaneously, and location information was recorded with GPS integrated into the B field meter. Measurements commenced when the bus departed from the first stop and concluded when it reached the last stop. Measurements were taken from a height of approximately 1.5 m above the floor of the bus, with each measuring device stabilized on a tripod during the process. The data recorded on the device for each measurement were saved to a computer at the end of the session for analysis. The collected E and B field values were then compared with the exposure reference levels established by ICNIRP for both occupational and general public health.

### 2.3. Magnetic Field and RF-EMF Data Acquisition

In this study, magnetic field measurements were taken using the SMP2 field strength meter [33]. The SMP2 is a sophisticated instrument designed for measuring EMF across a wide range of frequencies. It offers 3 functionalities: broadband measurements (DC to 60 GHz), frequency spectrum analysis (DC to 400 kHz), and static field measurements. The SMP2 is capable of measuring E, H, and B fields. It allows users to view real-time data and analyze results directly on the device. The SMP2 includes internal memory for data logging (log time is configurable from 0.5 s to 6 min.), enabling users to store measurement data (instantaneous, max., min., and average) for later analysis. The device also supports accessories such as GPS modules for geolocated measurements. Furthermore, the SMP2 is designed to comply with international standards for EMF measurements, such as those set by the ICNIRP and the Institute of Electrical and Electronics Engineers (IEEE).

**Figure 4:**
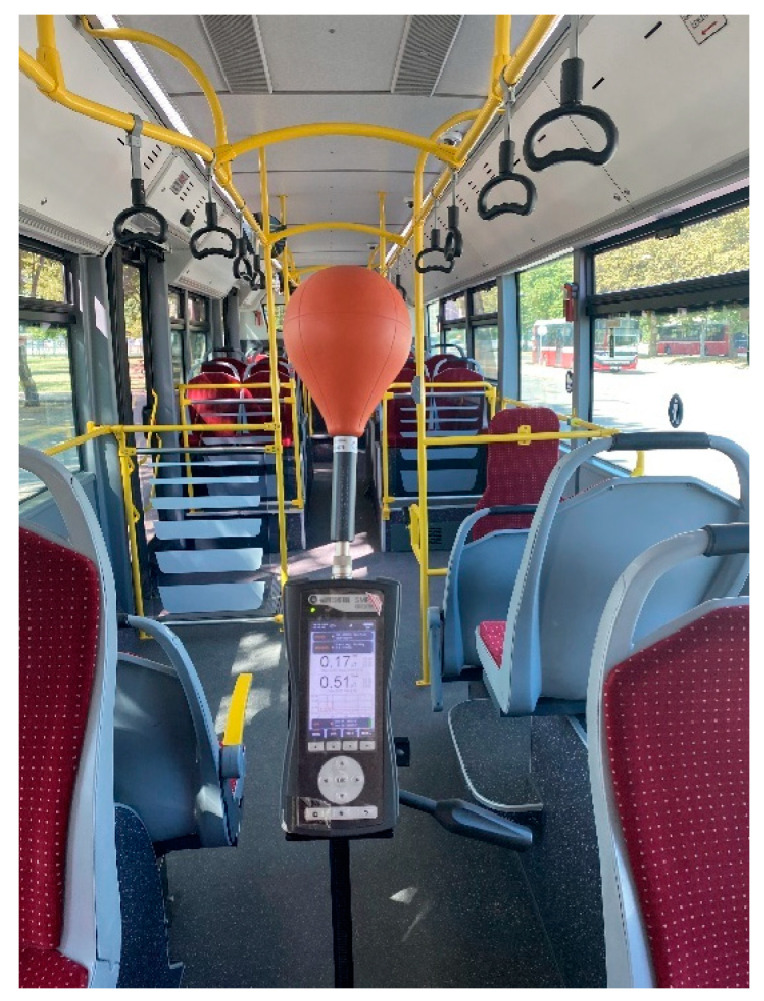
An example image of a measurement taken with the SMP2 inside the electric bus.

This study measured the B using an SMP2 field strength meter with a WP400 probe, covering a frequency range of 1 Hz to 400 kHz. The measurement range of the WP400 probe is 50 nT to 10 mT, with a resolution of less than 0.01 nT and a noise level below 50 nT. The graphical display shows root mean square (RMS) values, axis values, and average, maximum, minimum, and peak levels. The log time was set to 0.5 s, and both RMS and peak values were recorded. An example of a measurement taken with SPM2 inside the bus is shown in Figure 4.

The RF-EMF measurements were conducted using the EME SPY Evolution EMF meter, a device designed to continuously monitor human exposure to EMF across user-defined frequency bands ranging from 80 MHz to 6 GHz [34]. This range covers most of the RF spectrum, including frequencies used by mobile phones, Wi-Fi, Bluetooth, radio and television broadcasting, and other communication devices. The device measures the E field, providing information about the intensity of RF exposure in the environment. The EME SPY Evolution is a compact, lightweight device that is easy to carry and use in various settings.

One of the key features of the EME SPY Evolution is its ability to monitor multiple frequency bands simultaneously. This capability is particularly useful in environments where various RF sources are present, as it allows users to assess the contribution of each source to the overall RF exposure. The device has internal memory for data logging, allowing users to store measurement data for later analysis. The EME SPY Evolution is designed to comply with international standards for RF exposure assessment, such as those set by the ICNIRP and other relevant regulatory bodies.

**Figure 5:**
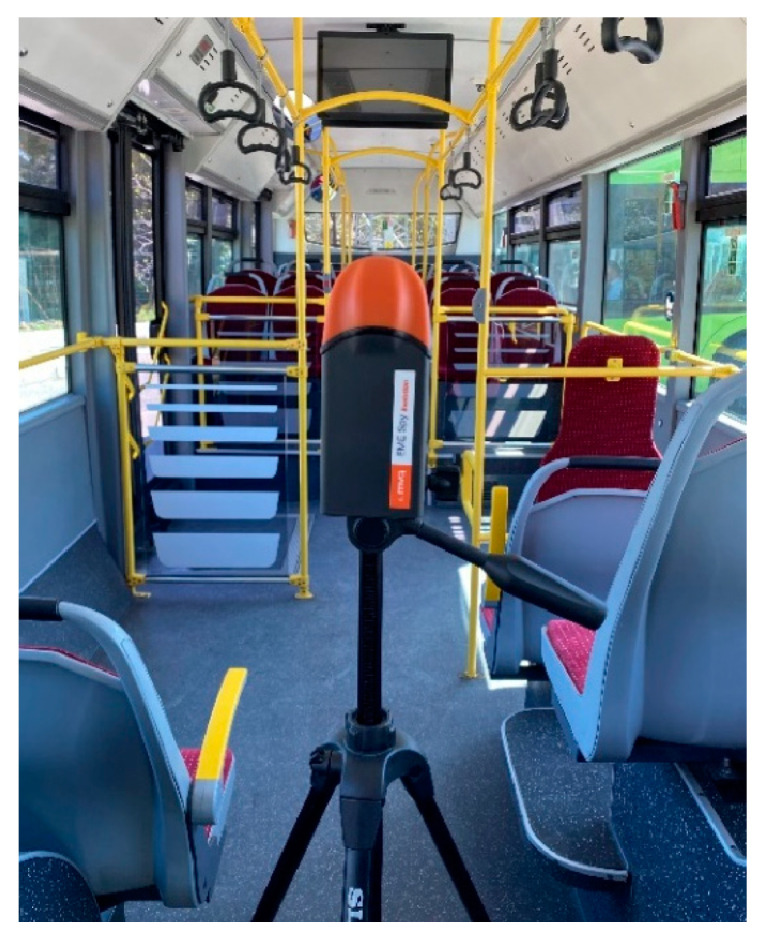
An image of a measurement being taken with the EME SPY inside an electric bus.

Equipped with a tri-axial E-field probe within the frequency band of 80 MHz to 6 GHz, the EME SPY offers high sensitivity, with readings of 0.05 V/m in the 80 MHz to 0.7 GHz and 3 GHz to 6 GHz ranges and 0.02 V/m in the 0.7 GHz to 3 GHz range. The EME SPY Evolution can record up to 166,000 samples in a 20-band scenario, with a customizable recording period ranging from 2 to 255 s, depending on the desired scenario. This study adjusted the measurement intervals to 6 s to effectively capture the time-based RF-EMF characteristics. For detailed technical specifications of the EME SPY, please refer to [32]. An example of a measurement taken with the EMS SPY in the bus is shown in Figure 5. The measured frequency bands of the EME Spy Evolution are summarized in Table 2.

Twenty channels of band-selective RF-EMF, recorded at 6 s intervals for 120 min, were used in the analyses using EME SPY in this study. The exposure levels in RMS are collected from 20 different RF-EMF frequency bands, and the total RF-EMF is referred to as the sum of all frequency bands. The total E (E_T,r_) level at r^th^ sample in the environment is calculated using Equation (1) for band-selective RF-EMF measurements. E_i,r_ is the electric field strength for the i^th^ band of r^th^ sample, and N refers to the frequency spectrum’s total number of bands (the value of N is 20).

(1) $$E_{T,r}=\sqrt{\sum_{i=1}^{N}{\left(E_{i,r}\right)}^{2}}$$

For given i^th^ band, the resulting isotropic E field strength of the three directions (E_i,r,x_, E_i,r,y_, and E_i,r,z_ are the electric fields measured in the x, y, and z directions of r^th^ sample) can be expressed as follows (2):

(2) $$E_{i,r}=\sqrt{{\left|E_{ix,r}\right|}^{2}+{\left|E_{iy,r}\right|}^{2}+{\left|E_{iz,r}\right|}^{2}}$$

We are considering a six-minute average according to ICNIRP’s guidelines. The time-averaged electric field strength (E_T,avg_) is calculated as follows (3):

(3) $$E_{T,avg}=\sqrt{\frac{\sum_{r=1}^{R}\left(E_{T,r}\right)}{R}}$$

where R denotes the total number of samples in a time window for an average RF-EMF value. For example, in the case of 6 min, value of R is 60 considering data are taken every 6 s. The contribution of the predominant bands to the total E is calculated using (4) as follows:

(4) $$P_{r}=\frac{E_{i,r}^{2}}{E_{T,r}^{2}}\times 100$$

P_r_ shows the percentage of a band (E_i,r_) relative to the total E.

This study analyzed all data using an Intel Core i7-12650H 2.30 GHz, 16.0 GB RAM computer, and MATLAB version 2022b. For RF-EMF measurements, each RF-EMF value was recorded at three different locations on each bus across two different bus routes during morning and evening hours and was analyzed using MATLAB software, version 2022b. The total RF-EMF values and band selective RF-EMF values were extracted from the data for analysis. Using these values, the variation in RF-EMF during the measurement period, box plots, and the contribution of each band to the total RF-EMF were analyzed. Additionally, using the GPS data and total RF-EMF values recorded during the measurements, the data were mapped and visualized with the help of ArcGIS software, version 10.1.

Similarly, B measurements were performed at the same locations and times as the RF-EMF measurements, resulting in a total of six sets of measurement data. Using these values, the variation in B during the measurement period, box plots, and statistical analyses of B measurements were conducted. The B values were also mapped and visualized. In addition to these measurements and assessments, B measurements were also taken during charging and for both normal and electric buses. Anderson–Darling goodness-of-fit tests were performed on all RF-EMF and B measurement data to ensure the statistical validity of the analyses.

### 2.4. Magnetic Field Measurements during Charging

**Figure 6:**
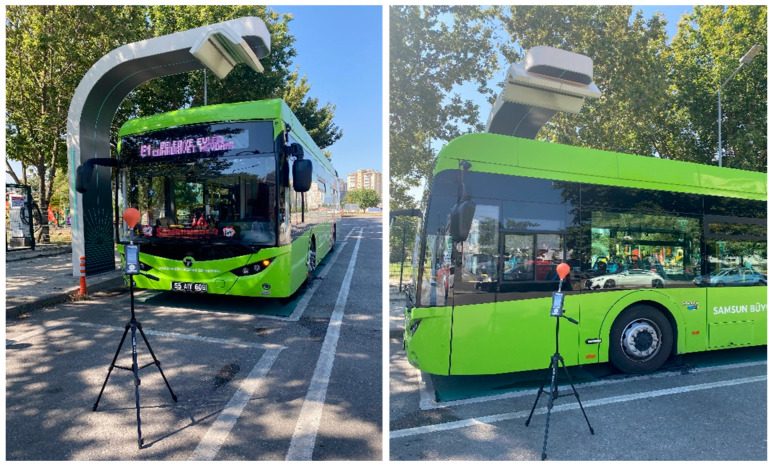
Photographs of B measurements during charging.

Photographs of the locations of B measurements made outside and inside the bus during charging are given in Figure 6.

**Figure 7:**
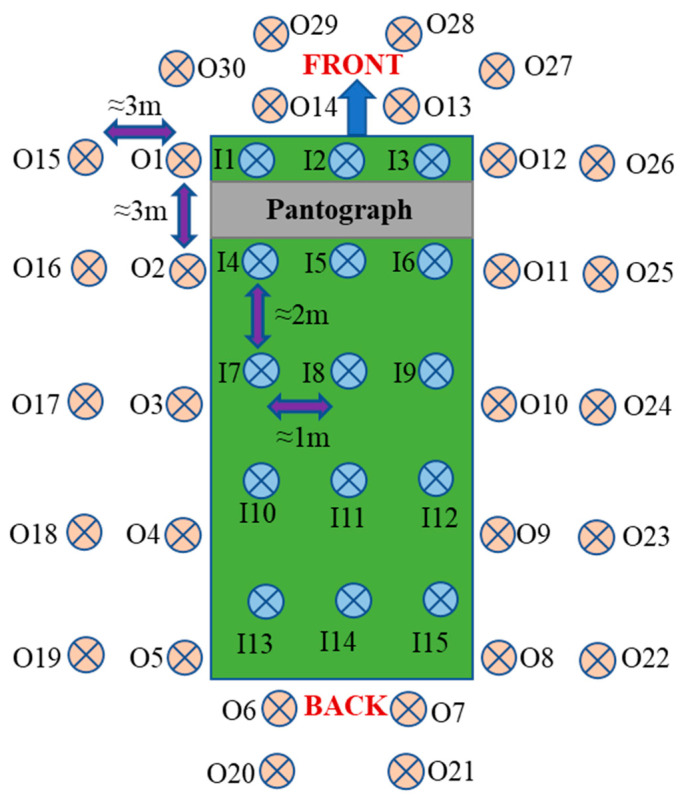
Locations of *B* measurements taken outside and inside the bus during charging (O is outside, and I is inside).

A visualization of the measurement subjects is given in Figure 7. Figure 7 also shows the approximate distances between the measurement points outside and inside the bus.

### 2.5. Anderson–Darling Test

The Anderson–Darling (AD) test [35,36], a modification of the Kolmogorov–Smirnov test, is employed to assess which statistical distribution best aligns with the sample data. This test measures the distance between the expected distribution and the empirical cumulative distribution function, focusing on detecting deviations in the distribution’s tails. The Anderson–Darling test is particularly robust in identifying deviations from normality due to its lower sensitivity to outliers compared to other tests.

In the AD test, the decision to reject or not reject the null hypothesis is based on a comparison between the test statistic and a critical value, which can be derived analytically or from the asymptotic distribution of the test statistic for each distribution. The test evaluates two hypotheses: assuming the data follow a specific distribution and positing otherwise. The outcome relies on two key metrics: the *p*-value and the critical value. A low *p*-value (typically below 0.05) or a test statistic that exceeds or equals the critical value suggests that the data do not conform to the specified distribution.

Previous studies have effectively utilized the Anderson–Darling test to ascertain appropriate statistical distributions for modeling electric field strength in various settings, such as indoor environments and electrically large enclosures. Similarly, in this study, we employ the Anderson–Darling goodness-of-fit test to identify the best-fitting distributions for the measured B and RF-EMF levels. The distributions considered include the normal, generalized extreme value (GEV), lognormal, t-location scale, Weibull, and loglogistic distributions.

## 3. Magnetic and RF-EMF Measurement Results

The magnetic and RF-EMF measurement analyses were conducted for two distinct electric bus routes in Samsun, at three different locations within each bus, during both the morning and evening hours (in the rest of this paper, the morning measurements will be denoted by “m” and the evening measurements by “n”). These are comprehensively examined below. In addition, while the electric bus is being charged, the B changes outside and inside the bus are measured and analyzed.

### 3.1. Measurement Results for E1 Route

**Figure 8:**
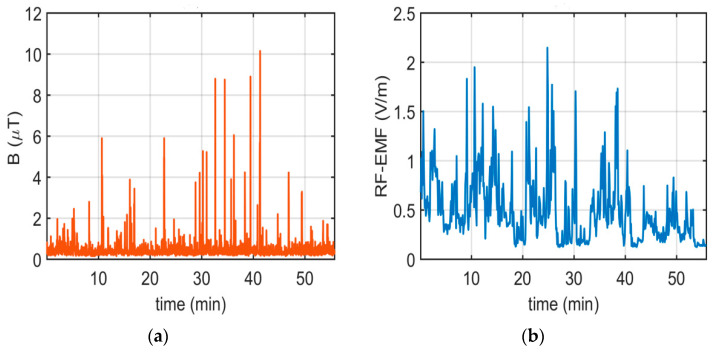
Results of (**a**) B and (**b**) RF-EMF measurements taken in the morning in the driver’s area on the E1 route.

For the E1 route, Figure 8 illustrates the variation in B and RF-EMF values measured at the driver’s position during the morning hours (L1_n) along the route over 56 min. As depicted in Figure 8a, there are instances of sudden changes in B measurements.

Variations in B were observed along the electric bus route, influenced by factors such as the bus’s location on the route, road slope, and passenger load. The B level in the electric bus measurements increases during specific operational phases, including acceleration, braking, descending a slope, and ascending a hill. An increase in the number of passengers and a positive road slope draw more current from the power line, resulting in higher B values inside the electric bus. Additionally, during braking, the B value inside the bus was more than that during normal travel, attributed to the reverse brake current. These abrupt changes can be attributed to specific operational phases, including acceleration, braking, descending a hill, and ascending a hill, which increase the magnetic field level in electric bus measurements. Higher magnetic field levels were observed during the acceleration and deceleration phases, primarily attributed to traction motor operation.

Similarly, electric field levels were elevated near power conversion systems, such as inverters and battery packs. A statistical analysis revealed that passenger seating locations closer to the bus’s rear and sides experienced higher EMF exposure levels than those seated in the front section.

The highest recorded B during the measurement period (L1_n) was 10.1500 µT, with an average value of 0.4770 µT. Upon analyzing the RF-EMF measurement results, variations along the route are observed. The highest recorded RF-EMF value is 2.1465 V/m, with an average value of 0.4910 V/m.

**Figure 9:**
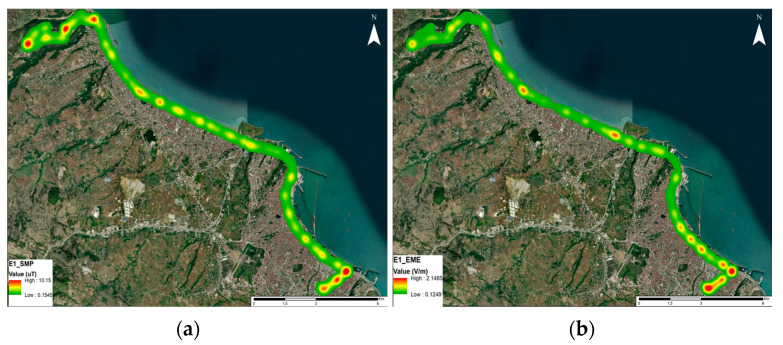
Map transfer for (**a**) magnetic field and (**b**) RF-EMF measurement results on the E1 route.

The measurement results were mapped and visualized, as depicted in Figure 9. In Figure 9a, areas with elevated B values correspond to the bus stops where the buses commence and conclude their journeys. Likewise, areas with heightened RF-EMF values indicate the presence of multiple base station locations.

**Figure 10:**
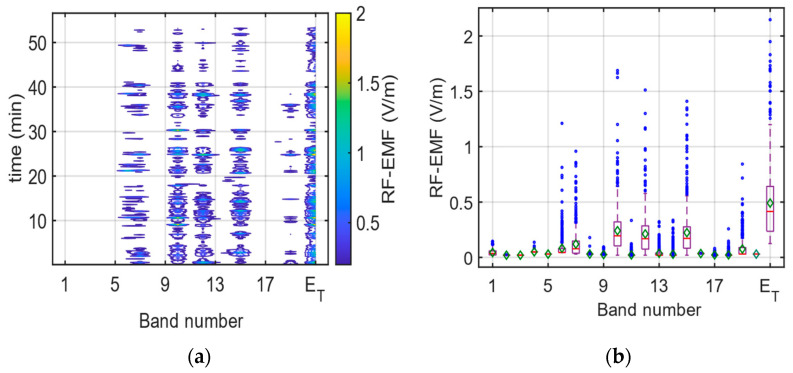
(**a**) Change in bands and (**b**) box plot display during band-selective measurements on the E1 route.

During the journey, the band-selective EMF results are given in Figure 10a and the box plot for each index is given in Figure 10b. In Figure 10b, the red line indicates the median value, while the green diamond and the blue circle indicate the average and the outlier value, respectively. As can be seen from Figure 10a, bands 6 (B28), 7 (LTE 800), 10 (GSM + UMTS 900), 12 (GSM 1800), 15 (UMTS 2100), and 19 (LTE 2600) have the highest contributions to the total RF-EMF value. This exposure behavior is explained by the EMF exposure caused by the base station downlink bands.

**Figure 11:**
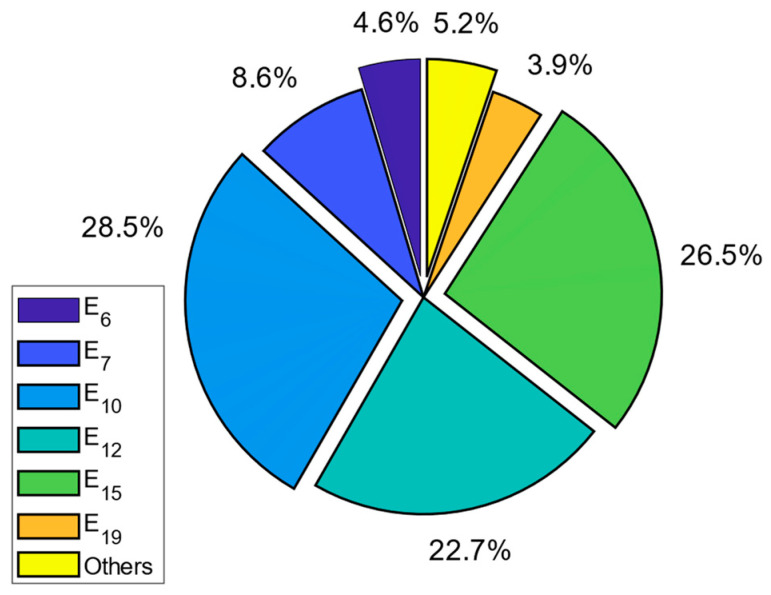
Contribution of bands to the total RF-EMR for L1_n measurements on the E1 route.

The contributions of these bands to the RF-EMF measurements are given as a pie chart in Figure 11. As can be seen from the figure, the band with the highest contribution to RF-EMF is band 10, with 28.5%. The next highest contribution is for band 15 with 26.5%.

**Figure 12:**
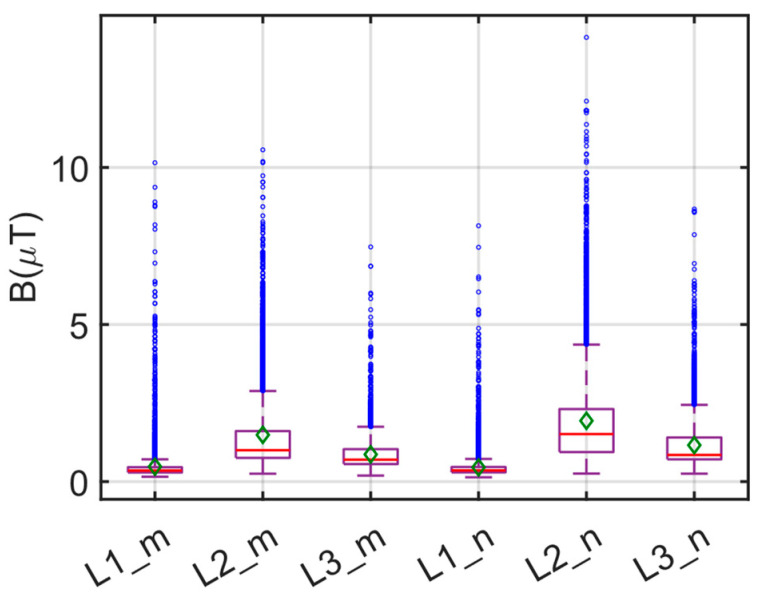
Box plot of B measurements for E1 route.

The box plot of the measurements made at the three locations and during the morning/nighttime hours for E1 is given in Figure 12. In Figure 12, the red line indicates the median value, while the green diamond and the blue circle indicate the average and the outlier value, respectively. Statistical evaluations of these measurements are presented in Table 3. When evaluating both Figure 12 and Table 3 concurrently, it becomes apparent that the B values recorded during the morning and evening periods are comparable. The highest B values were observed at the center of the bus, while the lowest values were recorded in the driver’s area. Specifically, the highest B of 14.140 µT was measured at position L2 during the evening hours (L2_n). The average B in the driver’s area was 0.477 µT in the morning and 0.4519 µT in the evening, showing minimal variance. Conversely, in the middle section of the bus, the average morning measurement yielded 1.4870 µT, which increased to 1.9355 µT in the evening.

Similarly, the average B in the rear section increased from 0.8649 µT to 1.1584 µT in the evening hours. The significant increase in magnetic field values in the center region of the electric bus is attributed to the presence of battery blocks in this region. Additionally, due to the higher traffic density, the increased magnetic field values during the evening hours can be attributed to the increased bus activity, including braking, stopping, and starting. Overall, considering all the measurement results, the recorded magnetic field values are well below the ICNIRP limits (500 µT and 100 µT).

**Figure 13:**
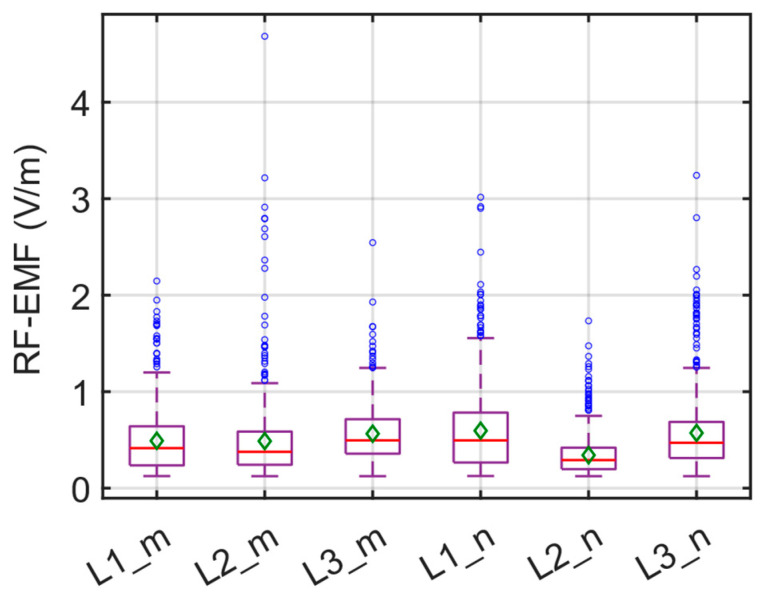
Box plot of the total RF-EMF measurements on the E1 route.

The box plot illustrating the total RF-EMF measurements conducted for the E1 route is presented in Figure 13. In Figure 13, the red line indicates the median value, while the green diamond and the blue circle indicate the average and the outlier value, respectively. As depicted in the figure, the average RF-EMF values hover around 0.5 V/m. The highest average RF-EMF value of 0.5952 V/m was observed at the driver’s position during the evening period. In contrast, the lowest value of 0.3418 V/m was recorded in the middle of the bus during the same timeframe. It can be inferred that these measured values (0.3418 V/m and 0.5952 V/m) remain well below the limit values provided by ICNIRP, verifying compliance with current EMF legislation, which constitutes 1.2–2.1% of the lowest reference levels (28 V/m) according to ICNIRP guidelines for the corresponding frequency band (80 MHz to 6 GHz).

**Figure 14:**
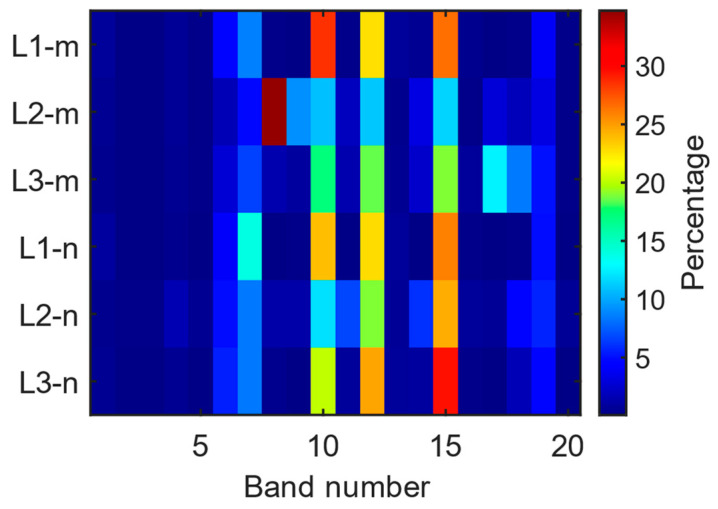
Contributions of bands to total RF-EMF measurements on the E1 route.

For the band-selective RF-EMF measurements, our evaluations depicting the contribution of each band to the total RF-EMF are presented in Figure 14. It is evident from the figure that bands 6, 7, 10, 12, 15, and 19 (base station downlink bands) contribute the most to the total RF-EMF. In the case of L2_m, there was a noticeable increase in the contribution of band number 8 (LTE 800, UL) in the measurement, with this band contributing 34.7% to the total RF-EMF. The total contribution from the downlink bands is 43.3%, while the uplink bands (5, 8, 9, 11, and 14) account for 51.3%. Unlike the other five band-selective measurements, it can be observed that in the L2_m measurement, the bands with the highest total RF-EMF contribution originate primarily from the uplink bands of the base station.

### 3.2. Measurement Results for E4 Route

**Figure 15:**
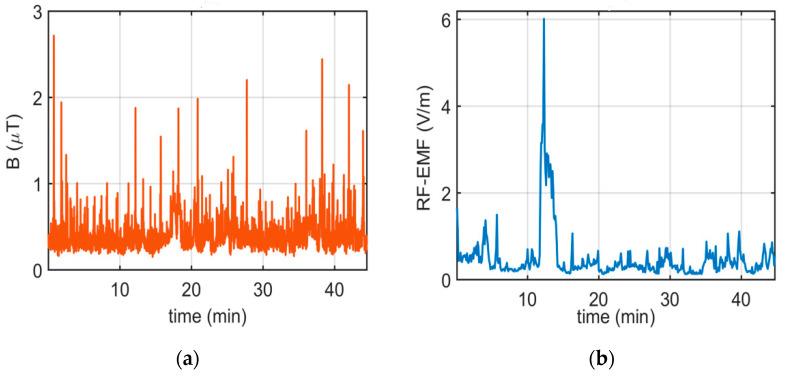
Results of (**a**) B and (**b**) RF-EMF measurements taken in the morning in the driver’s area on the E4 route.

For the E4 route, Figure 15 illustrates the variation in magnetic and RF-EMF values during the morning journey in the driver’s area. An analysis of the magnetic field measurements indicates a decrease in both the measured B values and peak values compared to the E1 route. The highest recorded B value was 2.7150 µT, with an average value of 0.4086 µT. Upon analyzing the RF-EMF values measured during this campaign, it is observed that while the values averaged below 1 V/m, they reached 6.01 V/m in one particular region.

**Figure 16:**
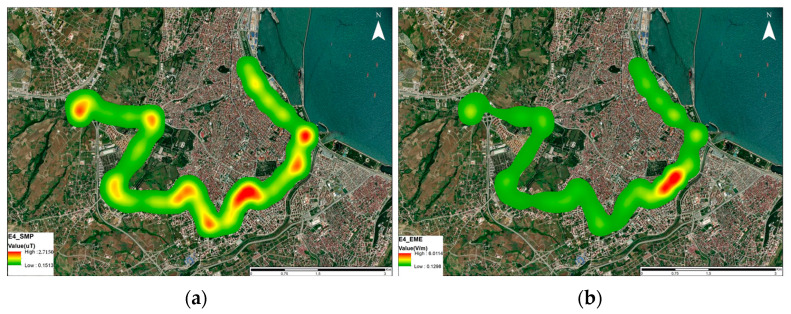
Map transfer for (**a**) B and (**b**) RF-EMF measurement results on the E4 route.

The B and RF-EMF values measured in the driver’s area on the E4 route were mapped to enhance visualization, as depicted in Figure 16.

**Figure 17:**
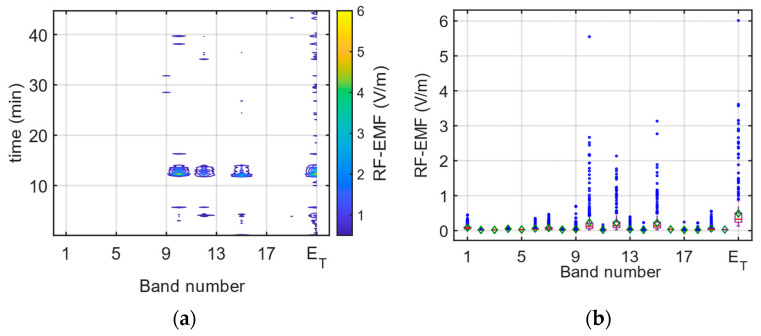
(**a**) Change in bands and (**b**) box plot display during band-selective measurements on the E4 route.

The band-selective RF-EMF measurements in the driver’s area on the E4 route are illustrated in Figure 17. In Figure 17b, the red line indicates the median value, while the green diamond and the blue circle indicate the average and the outlier value, respectively.

**Figure 18:**
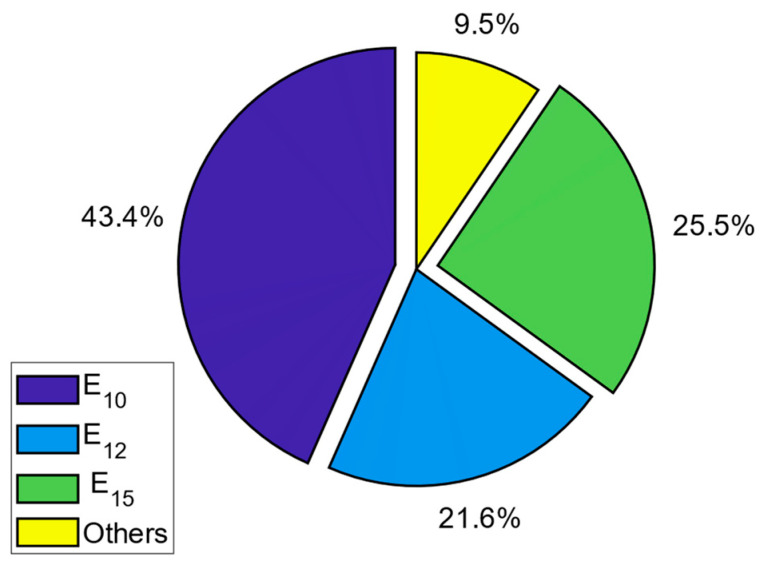
Contribution of bands to the total RF-EMR for L1_n measurements on the E4 route.

It can be observed from the figures that the bands contributing the most to the RF-EMF are bands 10, 12, and 15, corresponding to the downlink bands of the base stations. Among these bands, the highest contribution to the total RF-EMF is from band 10, constituting 43.4%. The contribution of the other bands to the total RF-EMF is presented in Figure 18 as a pie chart.

**Figure 19:**
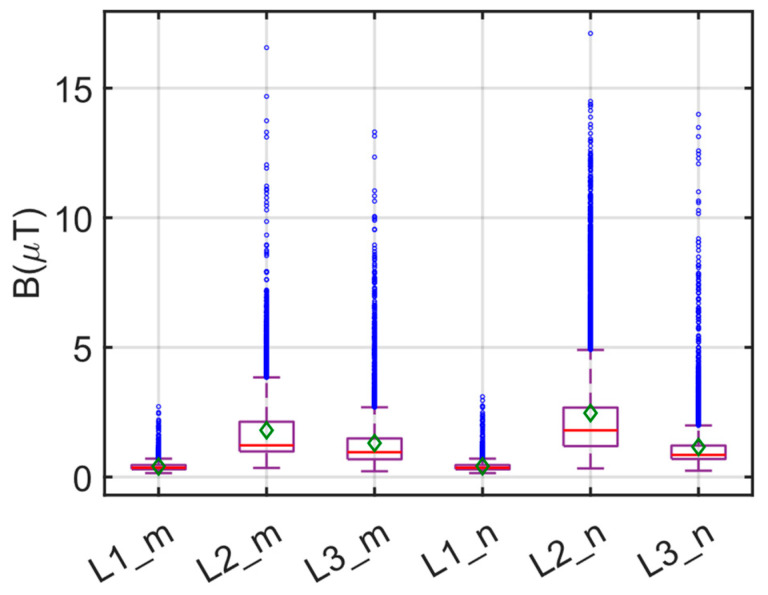
Box plot of B measurements on the E4 route.

A box graph illustrating the values of the B measurements conducted in the morning and evening in the remaining sections of the bus on the E4 route is presented in Figure 19. In Figure 19, the red line indicates the median value, while the green diamond and the blue circle indicate the average and the outlier value, respectively. Statistical analyses for the data are also provided in Table 4. Upon the simultaneous examination of Figure 19 and Table 4, considering the E1 route measurement outcomes, the lowest B value is observed in the driver’s area, whereas the highest value is detected in the center of the bus. The elevated values measured during the evening hours can be attributed to increased traffic density. Specifically, the highest average *B* value for the E1 route was 1.9355 µT at L2_n, while this value was recorded as 2.4663 µT for the E4 route.

**Figure 20:**
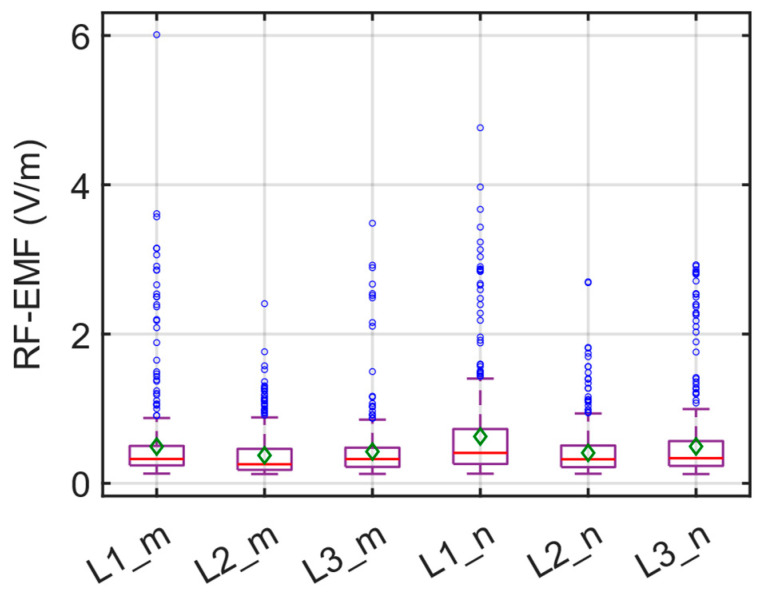
Box plot of the total RF-EMF measurements on the E4 route.

**Figure 21:**
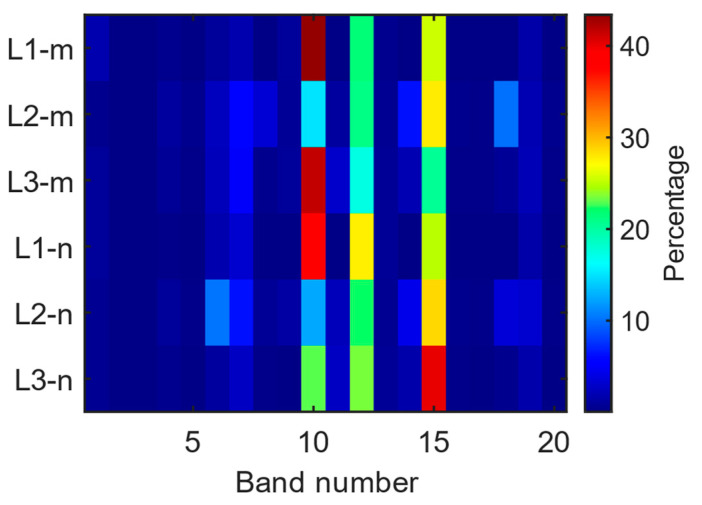
Contributions of bands to total RF-EMF measurements on the E4 route.

The results of six different RF-EMF measurements for the E4 route are depicted in Figure 20 as a box plot. In Figure 20, the red line indicates the median value, while the green diamond and the blue circle indicate the average and the outlier value, respectively. Overall, it can be observed that the measured average RF-EMF values are comparable, with a notable increase only in L1_n. Furthermore, upon an analysis of the contribution of the bands to the total RF-EMF, as illustrated in Figure 21, it is evident that the contribution of bands 10, 12, and 15 is significantly higher.

On the E4 route, there are also regular non-electric buses. Similar measurements for the E4 route were conducted for conventional buses, and the evaluations of their average B values are presented in Table 5. As observed from the table, there is not much disparity between the B values measured for non-electric buses. For the L1 location, the measurement results for both normal and electric buses are quite close, whereas significant differences are noted at the L2 and L3 locations. While the measured value for the electric bus at L2 is approximately four times higher than the normal value, this difference increases approximately 5.4 times during the evening.

### 3.3. Measurement Results for Electric Bus While Charging

**Figure 22:**
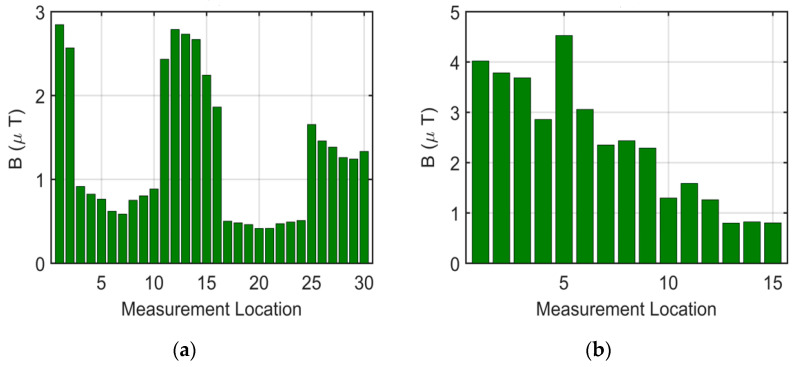
While the electric bus is charging, B values are taken from (**a**) 30 locations outside the bus and (**b**) 15 locations inside the bus.

While the electric bus was charging, B values were measured at 30 locations outside the bus and 15 locations inside the bus, as depicted in Figure 22. Before charging the bus, ambient B values were measured and recorded as 0.361 µT. As illustrated in Figure 22a, the B values were higher near the pantograph during the charging process, with lower values recorded at locations farther from the charging point. For the measurements taken off the bus, the highest B value recorded was 2.8452 µT, while the lowest was 0.4152 µT. For the measurements taken inside the bus, notably elevated B values were observed in the areas beneath the pantograph. The highest B value recorded inside the bus was 4.5260 µT, with the lowest being 0.7980 µT.

### 3.4. Statistical Analyses of Measurement Results

In this study, Anderson–Darling goodness-of-fit tests were performed for normal, GEV, lognormal, t-location scale, Weibull, and loglogistic distributions to determine the best-fitting distributions for the measured B and RF-EMF levels. The evaluation results for B are given in Table 6, and the RF-EMF results are presented in Table 7. When evaluating the measurement results of the E1 and E4 bus routes at three locations, the *p*-values for the normal distribution exceed 0.05. Similarly, for the other five distributions, the *p*-values also exceed 0.05. Consequently, each distribution in the table can represent the B value. Considering the lowest critical value, it can be observed that for the E1 route, the normal distribution exhibits a similar or lower critical value compared to the other distributions. For the E4 route, the lognormal distribution for L1 demonstrates the lowest critical value (closely followed by GEV). For L2 and L3, the GEV distribution demonstrates the lowest critical value. When Table 6 is evaluated as a whole, it becomes evident that the GEV distribution, exhibiting the lowest or closely comparable critical values, is most suitable for expressing the B measurements.

In the RF-EMF measurements, the *p*-value for the normal distribution at the L3 position in the attachment line is below 0.05. In contrast, the *p*-value exceeds 0.05 for all other conditions and distributions. Regarding the E1 route, the lowest critical value was observed for Weibull at L1, Weibull at L2, and loglogistic at L3. Conversely, for the E4 route, the lowest critical value was consistently observed for GEV across all three locations. Upon evaluating the RF-EMF measurements collectively, it is inferred that the GEV distribution, exhibiting the lowest critical value, can effectively express the RF-EMF for evaluations concerning critical values.

## 4. Conclusions

This study presents a detailed study of B and RF-EMF strength measurements within public electric buses, considering different routes and user densities under realistic conditions.

For the E1 route, variations in B were observed along the route, influenced by factors such as the bus location, road slope, and passenger load. An increased passenger density and positive road slope resulted in higher B values inside the electric bus, with additional increases observed during braking due to the reverse brake current. The RF-EMF measurements revealed variations along the route, with the highest recorded value reaching 6 V/m in certain areas, particularly in densely populated regions.

For the E4 route, a decrease in both the magnetic field intensity and RF-EMF values was observed compared to the E1 route, attributed to the E4 route’s straighter trajectory and shorter acceleration and braking times. Despite lower values, RF-EMF measurements still indicated potential exposure in certain areas, particularly in the city center with a dense base station presence.

The results of the RF-EMF measurements indicate a significant impact on user densities within the bus, with the increase in E-field exposure levels being more intensified for high-density cases. Exposure to RF-EMF inside the electric bus was found to originate from outdoor radiocommunication systems such as base stations. Band-selective RF-EMF measurements identified the downlink bands of base stations as the primary contributors to RF-EMF exposure in 11 measurements, while the uplink bands of base stations were the primary contributors in one measurement.

In the total RF-EMF measurements, the recorded values ranged from 0.124 V/m to 6.01 V/m, with average values ranging from 0.341 V/m to 0.6273 V/m. These measurements show RF-EMF strength levels (0.3418 V/m to 0.6273 V/m) that are far below the minimum limits (28 V/m for 80 MHz to 6 GHz) established by the ICNIRP standard, representing only 1.22% to 2.24% of these limits. The recorded B values ranged from 0.1360 µT to 17.1100 µT, with average values between 0.4086 µT and 2.4663 µT. Overall, the average B values remained well below the ICNIRP limit value of 200 µT for 50 Hz general public exposure, indicating compliance with current EMF legislation.

The results of the measurements taken while the buses were being charged indicated elevated B values near the pantograph, both inside and outside the buses, highlighting potential exposure during charging processes.

The results of the magnetic field intensity and RF-EMF measurements were subjected to the Anderson–Darling test, and it was concluded that the GEV distribution can best express all measurements.

This study enhances our understanding of EMF exposure in electric buses and its potential health risks. The findings offer valuable insights for developing strategies to reduce exposure and ensure safety in electric bus transportation. Specifically, this study identifies areas for optimizing EMF exposure for passengers and drivers, guiding design improvements, and creating EMF safety guidelines. The practical applications of the results obtained from this study are as follows: the identification of high-EMF areas enables targeted shielding or design modifications to improve the design of electric buses. Implementing measures to reduce EMF levels helps minimize the impact on passengers and drivers, thereby improving their safety and ensuring EMF levels meet local and international standards. Our study provides baseline data for further studies on EVs and general EMF exposure and facilitates more comprehensive analyses. In future studies, similar measurements are planned in different provinces, on other routes, and under various operating conditions to reveal the specific mechanisms of factors affecting B and RF-EMF measurements in electric buses, assess long-term effects, and develop EMF safety guidelines for electric buses.

## Figures and Tables

**Table 1:** A literature summary on low- and high-frequency measurements in public transport vehicles is presented.

Ref.	Environment/City	Device	Frequency
[13]	Tram, train, hybrid car/Melbourne	EMDEX II (Enertech Consultants, Campbell, CA, USA) EHP 50 (Narda Safety Test Solutions, Singen, Germany)	40 Hz–800 Hz 5 Hz–100 kHz
[14]	Electric tram bus/Malatya	SMP2 (Wavecontrol, Barcelona, Spain)	1 Hz–400 Hz
[15]	Electric tram/Bilboa	EME SPY 121 (Microwave Vision Group, Paris, France)	88 MHz–5850 MHz (2G, 3G, 4G, and 5G frequencies)
[16]	Hybrid cars/Varna	EMF 450 (Teledyne FLIR, Wilsonville, OR, USA)	1 Hz–1 MHz
[17]	Electric vehicle charges/Gyeongsan	NARDA EHP-50C (Narda Safety Test Solutions, Singen, Germany)	5 Hz–100 kHz
[18]	Electric train/Korea	HIOKI FT3470-50 Loop Antenna and Log Periodic antenna (HIOKI E.E. Corporation, Prefecture, Japan)	9 kHz–150 kHz 150 kHz–30 MHz 30 MHz–1 GHz
[19]	Metrobus and bus/Pamplona	EME SPY 121 (Microwave Vision Group, Paris, France)	88 MHz–5850 MHz
[20]	Urban electric transport vehicles/Warszawa	EME SPY 121 (Microwave Vision Group, Paris, France) MF THM1176-MF (Metrolab Technology, Geneva, Switzerland) EFA-300 (Narda Safety Test Solutions, Singen, Germany) EMDEX II (Enertech Consultants, Campbell, CA, USA)	88 MHz–2500 MHz 5 Hz–32 Hz 40 Hz–800 Hz
[21]	Bus, tram, train, metro/Toronto	TriField meter (AlphaLab, Salt Lake City, UT, USA)	30 Hz–500 Hz
[22]	Electric bus/Chattanooga	NARDA EHP-50D (Narda Safety Test Solutions, Singen, Germany) HI-3637 (ETS-Lindgren, Texas, USA)	5 kHz–100 kHz 2 kHz–400 kHz
[23]	Electric train and tramway/UK	EMDEX II (Enertech Consultants, Campbell, CA, USA)	
[24]	Tram/Samsun	PMM–8053 (PMM, Italy) NARDA SRM–3006 (Narda, Safety Test Solutions, Singen, Germany)	100 kHz–3 GHz 30 MHz–3 GHz

**Table 2:** Measured frequency bands of the EME Spy Evolution PEM.

Band Number	Technology	Frequency (MHz)
1	FM	87–107
2	TV3	174–223
3	TETRA I	380–400
4	TV4&5	470–615
5	B28 (UL)	703–748
6	B28 (DL)	758–803
7	LTE 800 (DL)	791–821
8	LTE 800 (UL)	832–862
9	GSM + UMTS 900 (UL)	880–915
10	GSM + UMTS 900 (DL)	925–960
11	GSM 1800 (UL)	1710–1785
12	GSM 1800 (DL)	1805–1880
13	DECT	1880–1900
14	UMTS 2100 (UL)	1920–1980
15	UMTS 2100 (DL)	2110–2170
16	B40TDD	2300–2400
17	WIFI 2G	2400–2483
18	LTE 2600 (UL)	2500–2570
19	LTE 2600 (DL)	2620–2690
20	WIFI 5G	5150–5850

DL: Downlink; UL: Uplink.

**Table 3:** Statistics of B measurements on the E1 route.

	B (µT)				
	Maximum	Minimum	Average	Median	Standard Deviation
L1_m	10.1500	0.1545	0.4770	0.3517	0.5693
L2_m	10.5600	0.2503	1.4870	0.9998	1.2609
L3_m	7.4700	0.1900	0.8649	0.6976	0.5306
L1_n	8.1440	0.1360	0.4519	0.3565	0.4033
L2_n	14.1400	0.2548	1.9355	1.5125	1.5277
L3_n	8.6750	0.2510	1.1584	0.8483	0.7550

**Table 4:** Statistics of B measurements on the E4 route.

	B (µT)				
	Maximum	Minimum	Average	Median	Standard Deviation
L1_m	2.7150	0.1513	0.4086	0.3592	0.1888
L2_m	16.5600	0.3487	1.8014	1.2210	1.3919
L3_m	13.3100	0.2213	1.3071	0.9562	1.1980
L1_n	3.0990	0.1502	0.4174	0.3564	0.2264
L2_n	17.1100	0.3326	2.4663	1.7990	2.1493
L3_n	13.9900	0.2429	1.1579	0.8522	1.0174

**Table 5:** Comparison of B values measured for electric and normal buses for the E4 route.

	Electric Bus B_avg_ (µT)	Non-Electric Bus B_avg_ (µT)
L1_m	0.4086	0.4038
L2_m	1.8014	0.4491
L3_m	1.3071	0.4218
L1_n	0.4174	0.4061
L2_n	2.4663	0.4542
L3_n	1.1579	0.4263
Overall	1.2598	0.4269

**Table 6:** Distribution test evaluations for B measurements.

Bus Route	Location	Parameters	Normal	GEV	Lognormal	T-Location Scale	Weibull	Loglogistic
E1	L1	*p*-value	0.9866	0.9795	0.9782	0.9773	0.9654	0.9557
E1	L1	critical value	0.2129	0.2312	0.2339	0.2360	0.2587	0.2744
E1	L2	*p*-value	0.1901	0.7681	0.4655	0.3368	0.1989	0.5715
E1	L2	critical value	1.4454	0.4772	0.8195	1.0384	1.4121	0.6826
E1	L3	*p*-value	0.8536	0.8391	0.8659	0.8339	0.8123	0.7943
E1	L3	critical value	0.3935	0.4081	0.3810	0.4131	0.4343	0.4518
E4	L1	*p*-value	0.9331	0.9519	0.9622	0.9245	0.7825	0.9269
E4	L1	critical value	0.3057	0.2801	0.2640	0.3164	0.4633	0.3135
E4	L2	*p*-value	0.0724	0.9840	0.5373	0.8608	0.0931	0.9478
E4	L2	critical value	2.1961	0.2200	0.7239	0.3862	1.9937	0.2859
E4	L3	*p*-value	0.0749	0.9852	0.2527	0.4192	0.0767	0.5283
E4	L3	critical value	2.1694	0.2167	1.2390	0.8897	2.1503	0.7352

**Table 7:** Distribution test evaluations for RF-EMF measurements.

Bus Route	Location	Parameters	Normal	GEV	Lognormal	T-Location Scale	Weibull	Loglogistic
E1	L1	*p*-value	0.7953	0.7505	0.7465	0.7641	0.8173	0.6384
E1	L1	critical value	0.4508	0.4944	0.4982	0.4811	0.4295	0.5610
E1	L2	*p*-value	0.3593	0.9956	0.9579	0.7972	0.4473	0.9846
E1	L2	critical value	0.9940	0.1768	0.2709	0.4490	0.8461	0.2184
E1	L3	*p*-value	0.8173	0.8355	0.6190	0.9925	0.7552	0.8834
E1	L3	critical value	0.4294	0.4116	0.6289	0.1927	0.4898	0.3628
E4	L1	*p*-value	0.1487	0.9913	0.5957	0.0943	0.3091	0.6593
E4	L1	critical value	1.6293	0.1975	0.6548	1.9833	1.0976	0.5858
E4	L2	*p*-value	0.3683	0.9130	0.8293	0.5198	0.4348	0.8844
E4	L2	critical value	0.9772	0.3301	0.4177	0.7460	0.8653	0.3617
E4	L3	*p*-value	0.0442	0.9990	0.2625	0.2612	0.0836	0.4977
E4	L3	critical value	2.6063	0.1458	1.2121	1.2155	2.0803	0.7749

## Data Availability

The data that support the findings of this study are available from the corresponding author upon reasonable request.
